# Brief Mindfulness Intervention for Family Caregivers of Persons With Dementia: A Pilot Study

**DOI:** 10.1093/geroni/igaa057.2455

**Published:** 2020-12-16

**Authors:** Hongdao Meng, Debra Dobbs, Lindsay Peterson, Punam Risal, Britney Veal, William Monaco

**Affiliations:** 1 University of South Florida, Tampa, Florida, United States; 2 University of South Florida, Winter Haven, Florida, United States

## Abstract

Family caregivers (FCGs) play a crucial role in helping persons with dementia age-in-place. This pilot study is designed to test a group-based mindfulness intervention for FCGs to reduce stress and enhance wellbeing. We recruited two groups of FCGs (n=7), one from an assisted living community and another from an adult day service center. We used a mixed-methods pre-post study design. Quantitative outcome measures included stress, sleep quality, and quality of life. We conducted focus group interviews to gain an in-depth understanding of the intervention acceptability. Participants reported that they benefited from participating in the intervention sessions, and were more aware of their reactions to stressors. FCGs indicated that the mindfulness practice assignments were acceptable and feasible. An unexpected finding was that the majority of the participants intended to continue with informal meetings after the pilot study. Implications of the intervention benefits for FCGs in the community will be discussed.

